# Delivery of Poorly Soluble Drugs via Mesoporous Silica: Impact of Drug Overloading on Release and Thermal Profiles

**DOI:** 10.3390/pharmaceutics11060269

**Published:** 2019-06-10

**Authors:** Tuan-Tu Le, Abdul Khaliq Elzhry Elyafi, Afzal R. Mohammed, Ali Al-Khattawi

**Affiliations:** Aston Pharmacy School, School of Life and Health Sciences, Aston University, Birmingham B4 7ET, UK; let3@aston.ac.uk (T.-T.L.); elzhryea@aston.ac.uk (A.K.E.E.); a.u.r.mohammed@aston.ac.uk (A.R.M.)

**Keywords:** mesoporous, poorly soluble drugs, solubility enhancement, solid dispersion, amorphisation, spray drying

## Abstract

Among the many methods available for solubility enhancement, mesoporous carriers are generating significant industrial interest. Owing to the spatial confinement of drug molecules within the mesopore network, low solubility crystalline drugs can be converted into their amorphous counterparts, which exhibit higher solubility. This work aims to understand the impact of drug overloading, i.e., above theoretical monolayer surface coverage, within mesoporous silica on the release behaviour and the thermal properties of loaded drugs. The study also looks at the inclusion of hypromellose acetate succinate (HPMCAS) to improve amorphisation. Various techniques including DSC, TGA, SEM, assay and dissolution were employed to investigate critical formulation factors of drug-loaded mesoporous silica prepared at drug loads of 100–300% of monolayer surface coverage, i.e., monolayer, double layer and triple layer coverage. A significant improvement in the dissolution of both Felodipine and Furosemide was obtained (96.4% and 96.2%, respectively). However, incomplete drug release was also observed at low drug load in both drugs, possibly due to a reversible adsorption to mesoporous silica. The addition of a polymeric precipitation inhibitor HPMCAS to mesoporous silica did not promote amorphisation. In fact, a partial coating of HPMCAS was observed on the exterior surface of mesoporous silica particles, which resulted in slower release for both drugs.

## 1. Introduction

The most important properties of promising drug candidates for oral dosage form are aqueous solubility and intestinal permeability. Over 40% of drugs on the market are BCS class II and IV, which have low solubility. Furthermore, new chemical entities are even less soluble compared to marketed products with a projection of up to 70–90% of drug candidates in the pipeline suffer from low solubility [1]. Following oral administration, drugs must dissolve in gastrointestinal fluids in order to be absorbed into the systemic circulation and exert a therapeutic action. The formulation development of low solubility drugs (BCS class II and IV) faces a great challenge as these drugs are poorly absorbed and usually exhibit subsequent low or variable oral bioavailability [2].

The problem of low solubility can be addressed by using solubilisation techniques, namely solid dispersion systems, size reduction, salt formation, prodrug, liposomes, etc. Among those techniques, solid dispersions are preferred by the industry due to their practicality and low cost. This technique is mainly based on a so-called “amorphisation”, whereby the crystalline drugs are converted into their high energy amorphous form, which exhibits a superior solubility in comparison with that of the original ones [3]. Mesoporous materials, e.g., mesoporous silica, a subclass of solid dispersion systems, are considered highly effective for drug amorphisation due to their ability to achieve the spatial confinement of drug molecules within their nanometre-scale pore structure [4]. Shen et al. [5] suggested that drugs would exist in an amorphous state within the mesoporous silica if the pore size were smaller than 12 times the drug’s molecular size. Mesoporous carriers also offer formulation flexibility due to tunable pore size and surface area [6]. Furthermore, this approach has shown applicability for both existing poorly soluble drugs and drug candidates in the pipeline with various chemical structures. Utilising mesoporous silica to enhance the bioavailability of poorly soluble drugs has been successfully demonstrated in various clinical studies conducted on rabbits, dogs, and mice [7].

For loading a drug into mesoporous silica, there are several techniques, which can be categorised into two main approaches: solvent-free methods and solvent-based methods. Solvent-free methods are comprised of physical mixing followed by heating to melt the drug, co-milling between the drug and mesoporous materials, and using supercritical carbon dioxide. Although solvent-free loading methods offer apparent advantages, e.g., no requirement for checking the residual solvent in drug products and low environmental impact, these methods are still under investigation to exhibit better performance in terms of the loading efficiency and stability of thermolabile drugs. On the other hand, solvent-based approaches offer a practical and straightforward solution for drug amorphisation within mesoporous silica. Simply put, a drug is dissolved in a suitable solvent, e.g., ethanol, then mixed/impregnated with mesoporous silica. The solvent can be removed by appropriate drying techniques at the end of the process. There are various factors that influence drug loading into mesoporous silica, such as the type of solvent, drug load, accessible surface area, and the pore volume of the mesoporous silica. In general, solvent-based loading techniques, especially spray drying, produce drug-loaded mesoporous silica with a high loading efficiency compared to solvent-free techniques [6]. However, higher drug loadings above 30% (*w*/*w*) could lead to incomplete amorphisation, i.e., a small amount of crystalline drug will remain on the exterior surface of the generated particles [5]. Drug molecules can theoretically adsorb onto the silica surface of mesopores as a monolayer or multilayers, depending on the drug’s molecular dimension, accessible surface area, and pore size [8]. Dening and Taylor [9] studied ritonavir–loaded–mesoporous silica at various drug loads of a 25–150% monolayer surface coverage and found that drug release decreased significantly as the drug load increased. Therefore, it would be prudent to systematically investigate the impact of drug loading beyond monolayer surface coverage (overloading) on the thermal behaviour of drug within mesoporous silica. Furthermore, the addition of a precipitation inhibitor to the mesoporous silica has been previously investigated to overcome this recrystallisation challenge. Lainé et al. [10] found that hypromellose acetate succinate (HPMCAS), when combined with mesoporous silica, promoted a complete amorphisation of Celecoxib. However, the advantage of such ternary system combining mesoporous silica with drug and precipitation inhibitors still requires further study, to confirm whether poorly soluble drugs with different chemical natures and crystallisation tendencies can benefit from it.

Felodipine and Furosemide are BCS class II and class IV drugs, respectively. Felodipine is mainly absorbed in the small intestine [11], i.e., and alkaline pH environment, while the stomach is the favoured absorption site for Furosemide [12], i.e., acidic pH environment. Hence, Felodipine and Furosemide were selected as model drugs in this study to represent two scenarios after oral administration in drug release from mesoporous silica. The aim of this study was to investigate the impact of drug overloading within mesoporous silica at 100–300% of the theoretical monolayer’s surface coverage on the release behaviour and thermal properties of loaded drugs. This included investigating the release profiles of the model drugs after loading within mesoporous silica alone and in combination with HPMCAS. Thermal profiles and particle morphology were also studied to confirm the amorphous/crystalline nature of the drug within the carrier system and the presence or lack of surface crystals. These studies will help elucidate the link between theoretical drug load and important formulation properties, such as loading efficiency, release behaviour, and the nature of the drug within mesoporous carriers.

## 2. Materials and Methods

### 2.1. Materials

Felodipine (FELO) and Furosemide (FURO) were obtained from Discovery Fine Chemicals (Dorset, UK) and Chemical Point (Surrey, UK), respectively. Mesoporous silica Syloid^®^ XDP 3050 (specific surface area of 310 m^2^/g, average pore size of 22.4 nm, pore volume of 1.74 cm^3^/g) was kindly provided by W.R. Grace and Co. (Worms, Germany). Aqoat^®^ (HPMCAS) was a generous gift from Harke Pharma (Muelheim an der Ruhr, Germany). Sodium phosphate monobasic, sodium phosphate dibasic, sodium chloride, and sodium lauryl sulfate (SLS) were purchased from Sigma-Aldrich (Dorset, UK). Hydrochloric acid 37%, acetone and ethanol were purchased from Fisher Scientific (Loughborough, UK). Deionised water was produced by Milli-Q Integral system (Hertfordshire, UK).

### 2.2. Methods

#### 2.2.1. Preparation of Drug-Loaded Mesoporous Silica Particles

Theoretical drug load was calculated based on an assumption that drug molecules would adsorb to the surface area of mesoporous silica particles in a packing geometry that increases the bonding between drug molecules and silica surface, i.e., to maximise the contact surface [9]. The following equation described by Dening and Taylor [9] was used to calculate the drug load (%, w_drug_/w_Syloid_) to theoretically obtain monolayer adsorption (equivalent to 100% surface coverage) in mesoporous silica:

(1) $$Theoretical\;drug\;load\;at\;monolayer\;adsorption\;\left(\%,\frac{g}{g}\right)=\frac{SSA\times M_{w}\times 10^{20}}{SA_{M}\times N_{A}}.$$

*SSA*: Specific surface area of mesoporous silica (m^2^/g), e.g., 310 m^2^/g for Syloid XDP 3050 (in-house data measured by gas adsorption porosimetry).

*M_w_*: Molecular weight of model drug (g/mol).

**Figure 1:**
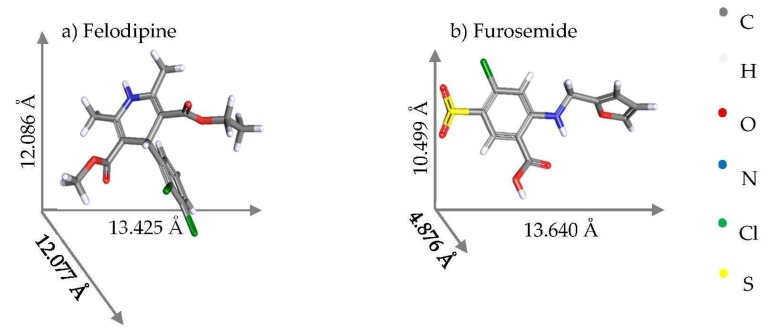
Molecular structures [13,14] and estimated molecular dimensions of Felodipine and Furosemide generated from the Cambridge Crystallographic Data Centre.

*SA_M_*: Maximum projected contact surface area of single molecule (Å^2^): calculated using the two largest molecular dimensions of drug molecule (Figure 1).

*N_A_*: Avogadro’s number (6.022 × 10^23^).

Syloid was added to either FELO or FURO in ethanol (10 mg/mL) to form suspensions at various theoretical drug loads (%, *w*/*w*): 10.8, 21.6, and 32.4% for FURO; 12.6, 25.2, and 37.8% for FELO to represent monolayer adsorption (100% surface coverage), double layer adsorption (200% surface coverage), and triple layer adsorption (300% surface coverage), respectively. The ternary Drug–Syloid–HPMCAS formulations were prepared in a similar fashion with the ratio of drug to HPMCAS at 10:1. Syloid was added to solutions of HPMCAS and either FELO or FURO in ethanol-acetone 50:50 (10 mg/mL). All of the suspensions were gently stirred for 12 h, then spray-dried at inlet temperature of 100 °C using a mini spray dryer Buchi B-290 and inert loop Buchi B-295 (Flawil, Switzerland) in closed mode with a nitrogen flow rate of 600 L/min, with a feed rate of 5 mL/min and a drying gas flow rate of 30 m^3^/h. Spray-dried FELO or FURO (prepared in the same procedure without the incorporation of either Syloid or HPMCAS), physical mixtures between Syloid and either FELO, or FURO with a ratio of 1:1 were used as control samples.

#### 2.2.2. Drug Loading Quantification

Drug loading within mesoporous silica samples was determined according to a TGA-based method described elsewhere [15,16,17]. Approximately 10 mg of drug–loaded mesoporous silica samples were placed into a platinum pan, then transferred to TGA instrument Perkin-Elmer Pyris 1 (Buckinghamshire, UK). The sample was treated with following temperature programme under a nitrogen flow of 20 mL/min: (1) heated from 50 to 120 °C and held for 5 min at 120 °C; (2) heated from 120 to 800 °C at a heating rate of 20 °C/min; and (3) held for 30 min at 800 °C. The drug load of FURO or FELO inside mesoporous silica was determined by the difference in weight loss between samples and blanks at a temperature range of 200–800 °C, whereby FELO or FURO were incinerated. The drug load was normalised to the surface area of the mesoporous silica to facilitate the comparison between studies using various types of mesoporous silica. Normalised surface area-based drug load and loading efficiency was calculated based on the two following equations.

(2) $$\mathrm{Surface}\;\mathrm{area}\;\mathrm{based}\;\mathrm{drug}\;\mathrm{load}\;\left(m^{2}/g\right)=\frac{\mathrm{Actual}\;\mathrm{drug}\;\mathrm{load}}{\mathrm{Specific}\;\mathrm{surface}\;\mathrm{area}\;\mathrm{of}\;\mathrm{mesoporou}\;\mathrm{silica}}$$

(3) $$\mathrm{Loading}\;\mathrm{efficiency}\;\left(\%\right)=\frac{\mathrm{Actual}\;\mathrm{drug}\;\mathrm{load}}{\mathrm{Theoretical}\;\mathrm{drug}\;\mathrm{load}}$$

#### 2.2.3. In Vitro Drug Release Studies

Dissolution testing was performed by using a USP I apparatus (rotating basket, 50 rpm) in an Erweka DT 126 dissolution tester (Heusenstamm, Germany). Each sample containing 20 mg of FELO was filled into a HPMC hard-shell capsule and tested in 500 mL of pH 6.5 medium with 0.25% SLS at 37 °C (adapted from USP 36 monograph with a reduction of SLS concentration from 1.0 to 0.25%). Samples were withdrawn during a 120 min period at the following timepoints: 15, 30, 60, 90, and 120 min. The concentrations of dissolved FELO were determined according to a HPLC method described in United States Pharmacopoeia [18] with mobile phase of pH 3 phosphate buffer:acetonitrile:methanol (30:45:25), column C18 (15 cm × 4.6 mm, 5 µm), flow rate of 1 mL/min, injection volume of 40 µL, and UV detector at 362 nm in an Agilent 1200 HPLC system (Santa Clara, CA, USA).

For FURO, samples containing 40 mg was filled into a HPMC hard-shell capsule and tested in 900 mL of a pH 3.0 medium with 0.25% SLS at 37 °C. The medium was prepared by dissolving 2 g of sodium chloride and 2.5 g of SLS in 400 mL of deionised water, then adding 0.1 mL of hydrochloric acid 37% and diluting with deionised water to 1000.0 mL [17]. The concentrations of dissolved FURO were determined using a HPLC method described elsewhere [19] with mobile phase of a phosphate buffer of pH 3: acetonitrile 60:40, C18 column, column temperature: 35 °C, flow rate of 1 mL/min, injection volume of 10 µL, and UV detection at 234 nm.

#### 2.2.4. Differential Scanning Calorimetry (DSC)

The thermal properties of samples were characterised by DSC instrument TA Q200 (New Castle, DE, USA). Each sample was accurately weighed (equivalently to 1 mg of FELO or FURO) into Tzero low-mass aluminium pan (sensitivity for a minimum sample size of 0.5 mg), and heated in the range of 50–250 °C (for FELO) or 100–300 °C (for FURO) at a scanning rate of 10 °C/min under nitrogen airflow of 50 mL/min. TA universal analysis 2000 software (version 4.5) was employed to analyse the resulting DSC thermograms.

#### 2.2.5. Scanning Electron Microscopy (SEM)

The surface of the drug-loaded mesoporous silica particles was examined by a Philips XL30 ESEM FEG (Hillsboro, OR, USA) operating at 10 kV under a high vacuum. Prior to SEM imaging, samples were coated with gold by a sputter coater. Approximately 1 mg of each sample was placed onto a double-sided adhesive strip on a sample holder. SEM images were taken at 2000× magnification.

#### 2.2.6. Statistical Analysis

Statistical analysis was carried out using GraphPad Prism 7.03 software. Statistically significant difference was considered at a *p* value < 0.05. All results are presented as mean ± standard deviation where applicable.

## 3. Results and Discussion

### 3.1. Thermal Profiles and Morphology of Drug-Loaded Mesoporous Silica

**Figure 2:**
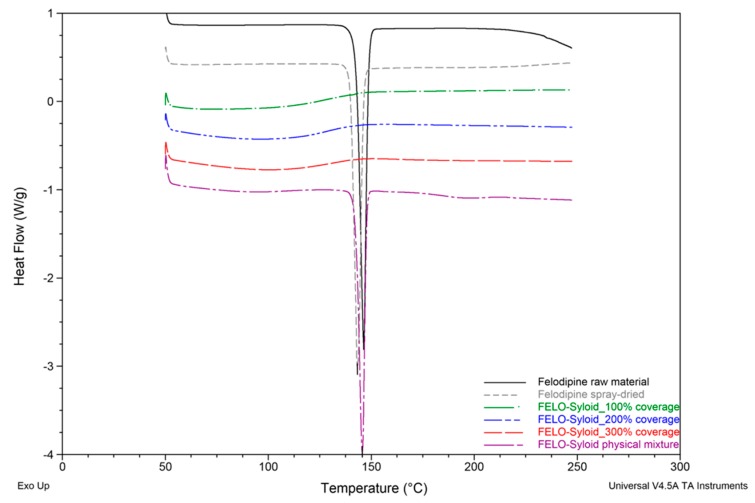
Differential Scanning Calorimetry (DSC) thermograms of Felodipine raw and spray-dried materials, Felodipine (FELO)-Syloid formulations at various drug loads, FELO-Syloid physical mixture.

**Figure 3:**
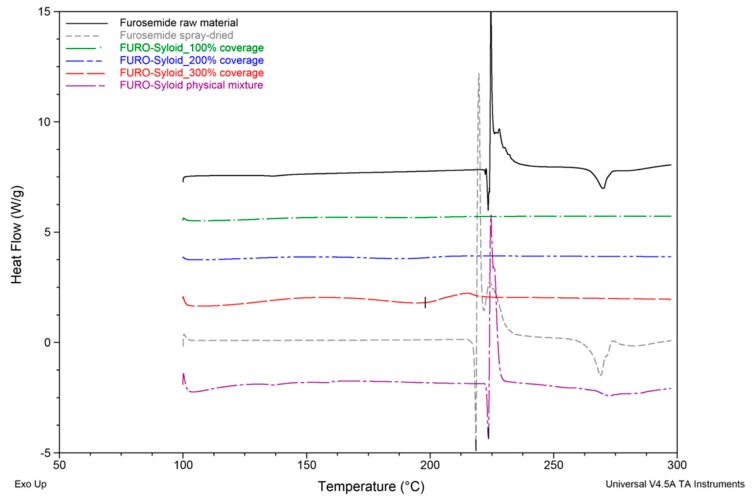
DSC thermograms of Furosemide raw and spray-dried materials, Furosemide (FURO)-Syloid formulations at various drug loads, FURO-Syloid physical mixture.

**Figure 4:**
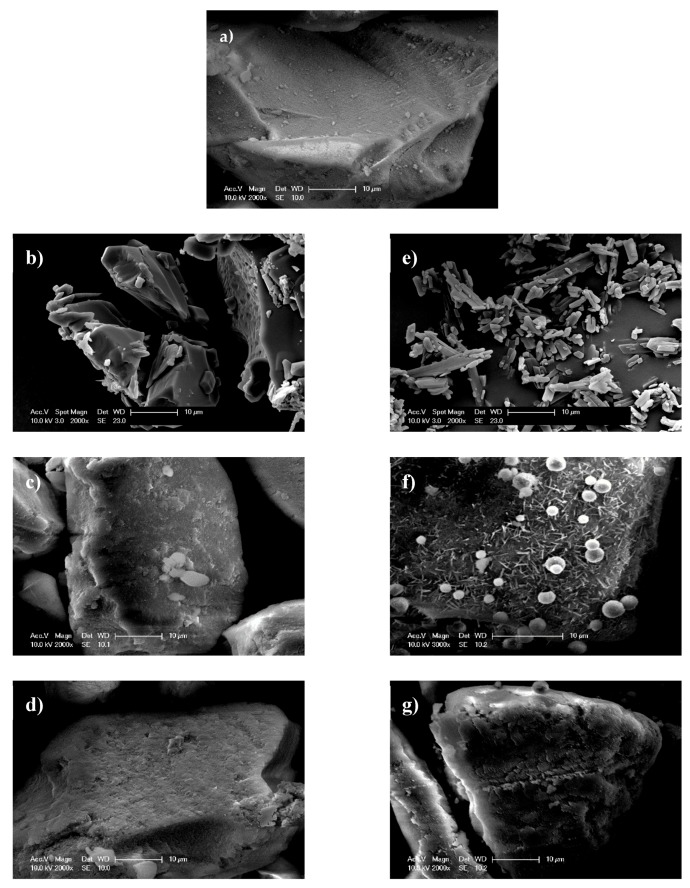
Particle surfaces of (**a**) mesoporous silica Syloid, (**b**) Felodipine raw material, (**c**) FELO-Syloid, (**d**) FELO-Syloid-hypromellose acetate succinate (HPMCAS), (**e**) Furosemide raw material, (**f**) FURO-Syloid, and (**g**) FURO-Syloid-HPMCAS. SEM images were taken for samples at a drug load of 300% surface coverage.

DSC analysis of Felodipine and Furosemide samples were presented in Figure 2 and Figure 3. Results revealed that Felodipine was completely converted to amorphous form inside mesoporous silica at all drug loads. This can be confirmed through the lack of melting peak of crystalline Felodipine (146.0 °C) in DSC thermograms of any Felodipine-Syloid formulations. In contrast, raw material and spray dried Felodipine (without mesoporous silica), exhibited sharp endothermic peaks at 146.0 ± 0.6 °C, 144.3 ± 1.8 °C, respectively, confirming their crystalline state [20], as can be seen in Figure 4b. The DSC data also indicated there was no interaction between Felodipine and Syloid in their physical mixture as there was no change in temperature (146.0 ± 0.5 °C) and the shape of the Felodipine melting peak.

Sharp endothermic peaks at 222.8 ± 0.8 °C and 223.9 ± 0.3 °C were observed in DSC curves of Furosemide raw material and FURO-Syloid physical mixture respectively (Figure 3), which is in agreement with the crystalline Furosemide in previous study [21,22]. This was further verified by SEM image (Figure 4e). The DSC data of physical mixtures between Syloid and Furosemide or Felodipine suggested that the drug still remains crystalline if deposited externally onto mesoporous silica particles, i.e., the physical mixture had no effect on amorphisation. Spray-dried Furosemide exists in crystalline form after the spray drying process, as confirmed through endothermic peak at 218.9 ± 0.7 °C. DSC analysis also revealed that Furosemide loaded within Syloid was completely amorphised at drug loads of 100% and 200% surface coverage as no endothermic peak was detected. However, at 300% coverage a broad endothermic peak was detected at a temperature of 198.7 ± 4.3 °C, indicating a small amount of crystalline Furosemide. In addition, this endothermic peak is shifted slightly to a lower temperature (198.7 °C) compared to that of raw material (222.8 °C), possibly due to the presence of nanocrystals. This result is consistent with a previous observation of Ibuprofen-loaded mesoporous silica [15], whereby researchers suggested that a nanocrystal form would cause a melting point shift. The formation of Furosemide nanocrystals at the highest drug load of 300% surface coverage can be observed in SEM image (Figure 4f). After drug loading, the surface of silica becomes rough as can be seen in FELO-Syloid (Figure 4c), particularly in FURO-Syloid with many surface crystallites in comparison with original surface of mesoporous silica, which is relatively smoother (Figure 4a).

**Figure 5:**
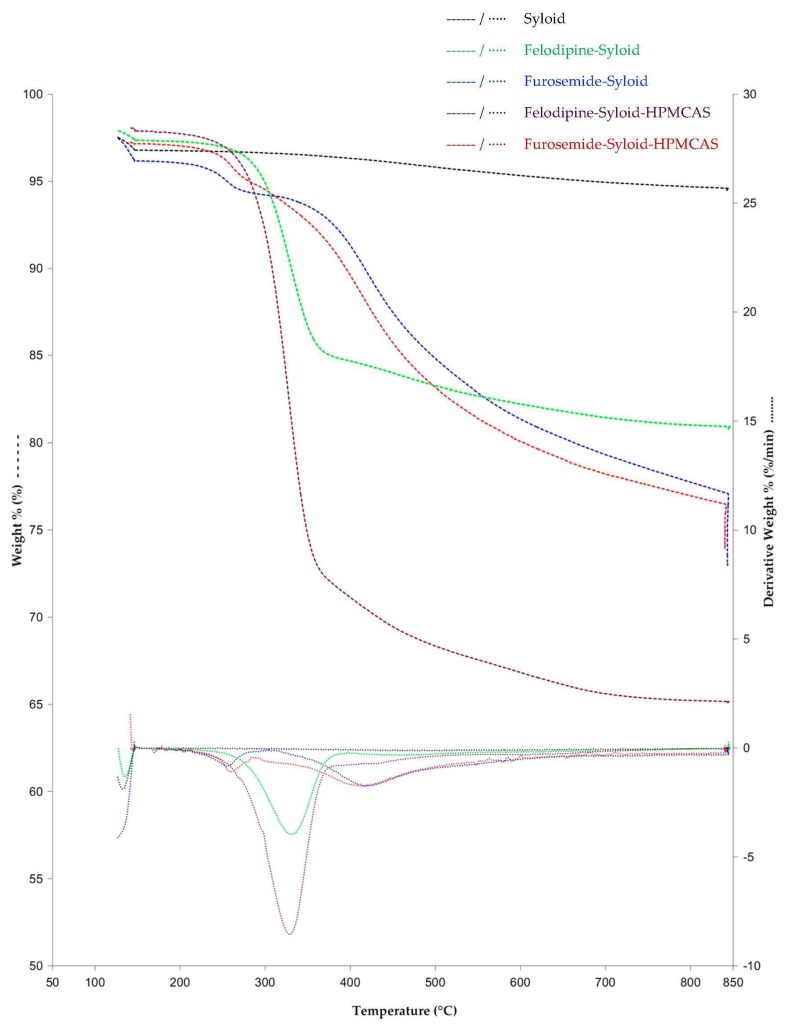
TGA weight% versus temperature and derivative weight% curves of Syloid, Felodipine-Syloid, Furosemide-Syloid, Felodipine-Syloid-HPMCAS, and Furosemide-Syloid-HPMCAS at a drug load of 300% surface coverage. Temperature programme under a nitrogen flow of 20 mL/min: (1) heat from 50 °C to 120 °C and held for 5 min at 120 °C; (2) heat from 120 °C to 800 °C at a heating rate of 20 °C/min; (3) held for 30 min at 800 °C.

TGA results (Figure 5) were used to determine the drug load and loading efficiency and complement the thermal events observed in the DSC data. TGA curves of the original Syloid as well as the drug-loaded Syloid revealed a small weight loss at 130.9 ± 0.6 °C, which can be attributed to bound water loss. There was no further significant weight loss in the original Syloid from 200 to 800 °C. TGA weight% versus temperature and 1st derivative curves of Felodipine-Syloid showed a substantial weight loss caused by the drug decomposition in the range 200–800 °C. Felodipine has one-step decomposition starting from 277.7 ± 0.8 °C and reach the maximum decomposition rate at 338.3 ± 5.7 °C, while Furosemide has a multi-step decomposition, which takes place between 200 and 800 °C with an onset temperature of 227.5 ± 3.4 °C. Thermal decomposition of both Felodipine and Furosemide are similar to the reported studies [22,23]. There was no notable difference in the shape of the TGA curves, as well as 1st derivative of the weight between the drug-loaded Syloid and ternary systems. This may have been caused by an overlap between the decomposition of drugs and that of the HPMCAS which happens in the range of 270 to 300 °C.

Actual drug loads and loading efficiencies of Felodipine and Furosemide in mesoporous silica are presented in Table 1. As expected, the actual drug load increased as the theoretical surface coverage increased. Loading into mesoporous silica could be explained by physical adsorption, which depends on the extent of the available surface area of adsorbent and the amount of adsorbate. The loading process carried out at a low drug load, i.e., a 100% surface coverage, resulted in maximum loading efficiency (99.2% for Furosemide and 101.9% for Felodipine) and complete amorphisation. However, at higher drug load at 300% surface coverage, the free surface area of the adsorbent (mesoporous silica) became lower as more drug was added, leading to a lower loading efficiency of 57.4% and 73.8% for Furosemide and Felodipine, respectively.

Previously, Ambrogi et al. [17] studied Furosemide-loaded mesoporous silica prepared by rotary evaporation and were able to attain an amorphous state Furosemide with SBA-15 silica at a drug content of up to 30.0% ± 0.2% (0.3 g of Furosemide per 0.7 g of SBA-15 mesoporous silica). Furosemide-Syloid in our study exhibited complete amorphisation with a drug load of up to 16.6 ± 0.3%, i.e., 0.166 g of Furosemide per 1 g of Syloid. However, it should be noted that the SBA-15 silica used by Ambrogi et al. [17] had a surface area which was 2.5 times larger than that of Syloid (791 m^2^/g vs. 310 m^2^/g, respectively). Hence, despite the apparent difference in weight-per-weight drug load, after normalisation based on surface area, there is no difference in weight-per-surface area drug load between the two studies (0.542 ± 0.004 × 10^−3^ g/m^2^ in comparison with 0.535 ± 0.010 × 10^−3^ g/m^2^).

For Felodipine-loaded mesoporous silica, there were two previous studies using solvent impregnation methods, in which silica had a specific surface area of 584 m^2^/g and 1051 m^2^/g with a drug load of 25% [24] and 18.3% [25], respectively. The amorphous Felodipine inside Syloid was obtained at a drug load of 21.7%. Similar to Furosemide, the weight-per-weight drug load of Felodipine-mesoporous silica was converted to weight-per-surface area to enable a like-for-like comparison between different studies. The Felodipine-Syloid in our study produced a higher weight-per-surface area drug load in comparison with those of the other two studies (0.697 × 10^−3^ compared to 0.428 × 10^−3^ g/m^2^ and 0.174 × 10^−3^/m^2^).

In general, the drug loading process for mesoporous particles is usually established on a case-by-case basis due to the differences in loading solvent, solubility, targeted drug load, surface area, pore size, and pore volume of the mesoporous materials being used. The use of co-spray drying in the current study as a solvent-based technique produced comparable or slightly higher drug loads to those reported in the literature using traditional solvent-evaporation. However, the real added advantage of using co-spray drying lies in the better drug loading efficiency and shorter processing time. During spray drying, there is a possibility that solute/drug diffusion towards the particle centre might facilitate drug entrapment inside the pores. The findings of higher drug loading efficiency via spray drying are also in agreement with previous studies [26,27].

### 3.2. In Vitro Drug Release from Mesoporous Silica

**Figure 6:**
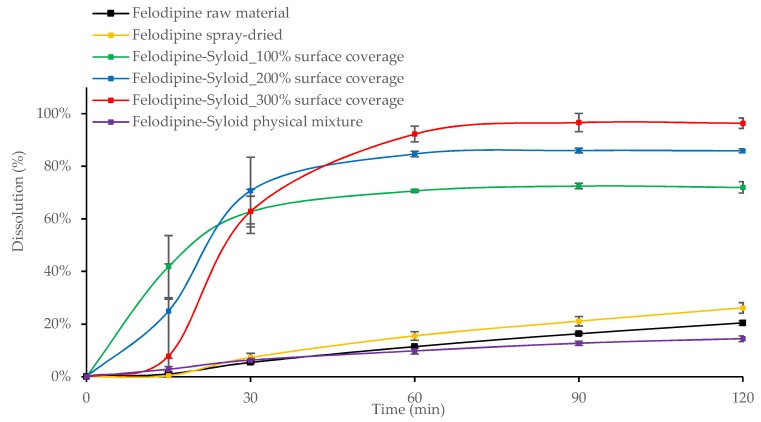
Release profiles of Felodipine raw and spray-dried materials, Felodipine-Syloid formulations at various drug loads, Felodipine-Syloid physical mixture. Testing conditions: phosphate buffer pH 6.5 + 0.25% SLS, 500 mL, USP apparatus 1, 50 rpm. The medium was adapted from a USP 36 monograph with SLS used to maintain a sink condition at the minimum possible amount.

**Figure 7:**
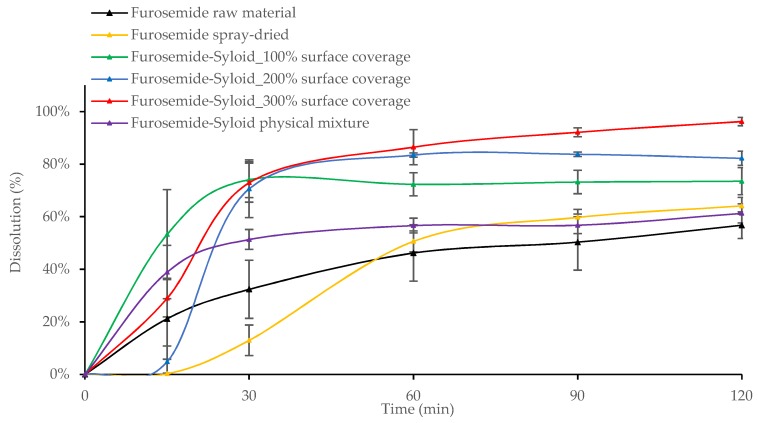
Release profiles of Furosemide raw and spray-dried materials, Furosemide-Syloid formulations at various drug loads, Furosemide-Syloid physical mixture. Testing conditions: HCL-NaCl medium pH 3 + 0.25% sodium lauryl sulfate (SLS), 900 mL, USP apparatus 1, 100 rpm. Medium was adapted from a study carried out by Ambrogi et al. [17] with the addition of SLS to maintain sink condition.

Results showed that after 30 min the dissolution of all Felodipine-Syloid or Furosemide-Syloid samples were much higher than that of the physical mixtures, both raw and spray-dried materials (*p* < 0.05). Felodipine exhibited a low dissolution within 60 min with only 9.8%, 11.4%, and 15.5% dissolution for the physical mixture, raw materials, and spray-dried materials, respectively, compared to over 70% dissolution in any Felodipine-Syloid samples (Figure 6). Similarly, within the first 60 min, the percentage of Furosemide released from mesoporous silica at 100%, 200%, and 300% of theoretical surface coverage reached 69.7%, 83.4%, and 86.5%, respectively while the physical mixture, the raw Furosemide and spray-dried materials, achieved only 56.6%, 46.2%, and 50.6% dissolution, respectively (Figure 7).

Although mesoporous silica is capable of enhancing the dissolution of Felodipine and Furosemide, a reversible adsorption between drug molecules and silica surface might happen. This result is in agreement with a previous study by Dening and Taylor [9], which investigated the release of ritonavir from mesoporous silica. The authors also found that drug release of ritonavir-loaded-mesoporous silica was incomplete. They suggested that there is a dynamic adsorption equilibrium between the drug adsorbed to mesoporous silica and the free drug dissolved in a dissolution medium. This dynamic equilibrium caused an incomplete drug release in dissolution testing, especially at a low drug load of 100% surface coverage (72.0% and 73.5% dissolution after 2 h for Felodipine and Furosemide, respectively). At this point, drug release from the drug-mesoporous silica samples reached a plateau, indicating that an adsorption equilibrium had been formed. However, as can be seen in our study, this dynamic equilibrium can be shifted in favour of more drug release via increasing the degree of drug load, i.e., silica surface coverage, possibly because the amount of drug adsorbed to the silica particles is negligible to that of the dissolved drug. Generally, the dissolution increased for both drugs, as the percentage of silica surface coverage increased. This is expected, as the higher amount of drug loaded inside the mesoporous silica released more drugs to the dissolution medium before reaching an adsorption equilibrium.

### 3.3. Influence of the Addition of HPMCAS to Mesoporous Silica on Drug Loading and Dissolution

In an attempt to increase loading efficiency and overcome the incomplete amorphisation at the highest drug load of 300% (in Section 3.1), HPMCAS was added to the drug-mesoporous silica to create a ternary system. The addition of HPMCAS to the Syloid resulted in increasing the Felodipine loading efficiency compared to that of the Syloid alone (*p* < 0.05). A weak basic drug Felodipine in a loading solution can partially dissociate to form a negatively charged anion, which might be converted into a conjugation with an acidic polymer HPMCAS. Such a conjugation between Felodipine and HPCMAS could enhance the loading efficiency as there could be an extra amount of Felodipine staying within the polymer matrix. However, there were no significant improvements in loading efficiency for Furosemide with the presence of HPMCAS (*p* > 0.05). This result could be explained because Furosemide is an acidic drug, and, therefore, no conjugation was formed between Furosemide and HPMCAS in comparison to the synergistic effect in drug loading between Felodipine and HPMCAS (Table 2).

**Figure 8:**
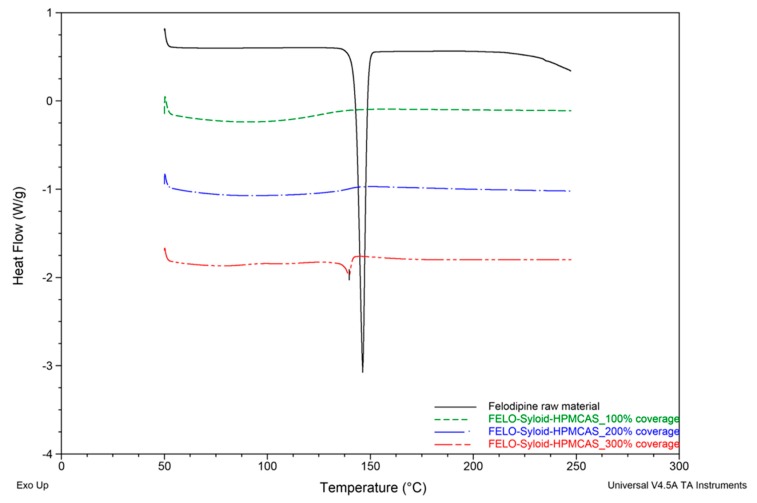
DSC thermograms of raw Felodipine material and FELO-Syloid-HPMCAS at various drug loads. Scanning rate: 10 °C/min. Scanning range: 50–250 °C.

**Figure 9:**
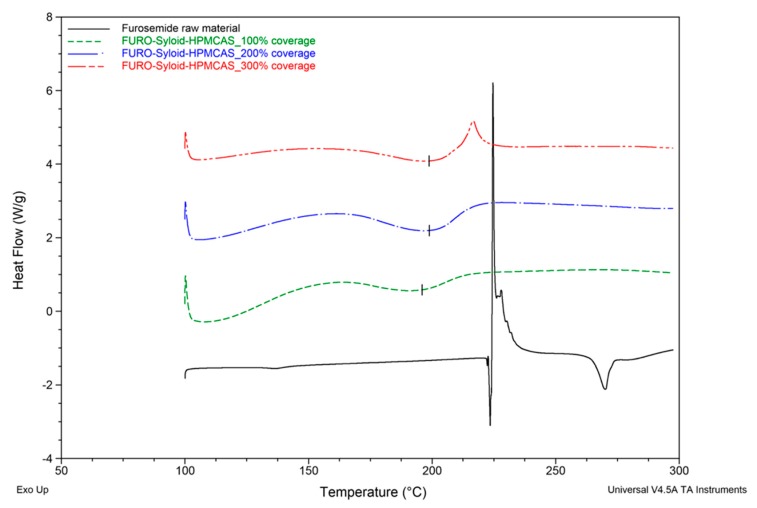
DSC thermograms of the raw Furosemide material and FURO-Syloid-HPMCAS at various drug loads. Scanning rate: 10 °C/min. Scanning range: 100–300 °C.

DSC results suggested that the addition of HPMCAS to the mesoporous silica did not result in the promotion of drug amorphisation. A small endothermic peak at 139.9 ± 0.2 °C was detected in the DSC thermogram of ternary Felodipine-Syloid-HPMCAS, at a drug load of 300% surface coverage (Figure 8). This indicated a small amount of crystalline Felodipine in the ternary system, although Felodipine-Syloid stayed amorphous in the absence of HPMCAS. The negative impact on amorphisation was clearly evidenced by the DSC data of the ternary Furosemide-Syloid-HPMCAS. Furosemide-Syloid at drug load of 100% or 200% surface coverage existed in an amorphous state (Section 3.1). Contrastingly, ternary Furosemide-Syloid-HPMCAS exhibited an incomplete amorphisation at all drug loads which was confirmed by broad endothermic peaks (Figure 9).

SEM images showed, to a certain extent, that HPMCAS might work as a coating polymer outside silica particles. In fact, a partial coating effect of HPMCAS as new surface ruggedness or layers could be seen in both Felodipine-Syloid-HPMCAS (Figure 4d) or Furosemide-Syloid-HPMCAS (Figure 4g), compared to the original surface of Syloid, which was relatively smoother (Figure 4a). The partial coating effect of HPMCAS was more apparent on the Furosemide-Syloid samples. There was a great number of Furosemide crystals on the external surface of the Furosemide-Syloid particles (Figure 4c), which was verified with the DSC result. Ternary Furosemide-Syloid-HPMCAS also contained crystalline Furosemide, as confirmed by DSC (Figure 9). However, there was no notable difference in the particle surface between Furosemide-Syloid and ternary Furosemide-Syloid-HPMCAS. Owing to the addition of HPMCAS, no surface crystals could be observed on the exterior of silica particles, possibly due to the layers of HPMCAS covering them (Figure 4g). SEM images, together with DSC data, suggested that HPMCAS might hinder the migration of drug molecules into the internal mesopores due to their coating effect, leading to more drug staying externally in silica particles.

**Figure 10:**
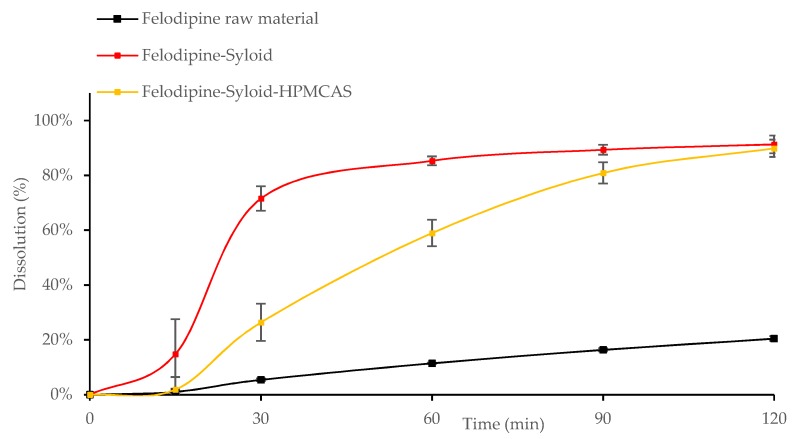
Release profiles of the raw Felodipine material, Felodipine-Syloid, and Felodipine-Syloid-HPMCAS at drug load of 300% monolayer coverage. Testing conditions: phosphate buffer pH 6.5 + 0.25% SLS, 500 mL, USP apparatus 1, 50 rpm.

**Figure 11:**
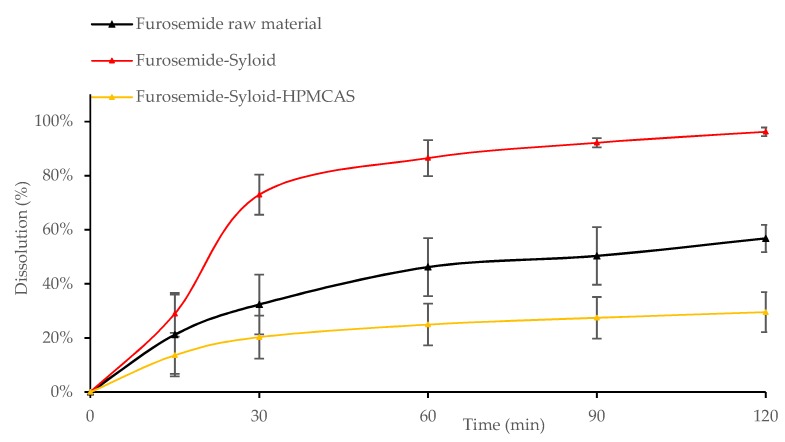
Release profiles of Furosemide raw material, Furosemide-Syloid, Furosemide-Syloid-HPMCAS at drug load of 300% surface coverage. Testing conditions: medium pH 3 + 0.25% SLS, 900 mL, USP apparatus 1, 100 rpm.

The inclusion of HPMCAS with Syloid did not help in enhancing the release rate or overall dissolution of Felodipine (Figure 10). In fact, the dissolution of Felodipine-Syloid-HPMCAS was significantly lower than that of Felodipine-Syloid within the first 90 min (*p* < 0.05). This polymer is only soluble at pH 5.5 or above, hence the partial coating of HPMCAS on the mesoporous silica was not dissolved at pH 3 acidic environment (optimal pH for Furosemide absorption) and resulted in an adverse effect on the drug release of Furosemide (Figure 11).

Furosemide-Syloid-HPMCAS still obtained 29% dissolution after 120 min as the drug was possibly released from silica particles through incomplete coating layers. A previous study [10] showed a more significant effect of HPMCAS on the dissolution of the poorly soluble drug celecoxib when loaded into the mesoporous silica Parteck (approximately a 5-fold solubility increase). The difference in the observation could be due to the different conditions, e.g., the dissolution medium of Tris buffered water pH 7.4 and a fasted state simulated intestinal fluid with pH 7.0 and the model drug Celecoxib used in their study. It is noticeable that the ratio of HPMCAS to Parteck silica was calculated as 14.7%, which was much higher than the 6.1% ratio of HPMCAS to Syloid silica in our study (calculated for Felodipine-Syloid-HPMCAS at a drug load of 300% surface coverage). Generally, a higher HPMCAS-to-silica particle ratio led to a thicker coating layer, if formed, and produced a more pronounced gastro-resistant effect, which could result in a delayed release effect in oral administration.

## 4. Conclusions

Syloid enhanced the dissolution of both model poorly soluble drugs, Felodipine and Furosemide, due to amorphisation within its mesoporous network. Increasing the drug load or percentage of theoretical surface coverage increased the maximum attained dissolution. However, overloading could lead to the formation of surface nanocrystals, as observed in the thermal and morphological studies. Incomplete drug release happened at all drug loads, which could be due to the reversible adsorption phenomenon mentioned in previous reports. This was predominant at the lower drug loads as less drug is available to dissolve before reaching an adsorption equilibrium. In addition, overloading resulted in a decrease of loading efficiency for both tested model drugs. A new drug load based on the drug amount to specific surface area of materials could be used in order to enable the comparison between various types of mesoporous materials. The addition of HPMCAS at a low concentration prevented the complete amorphisation of the drugs. Furthermore, it also slowed down the drug release due to partial coating, which formed on the exterior surface of mesoporous silica particles. In the future, this concept of combining mesoporous silica and polymers in hybrid structures will be further investigated for potential application in sustained release drug delivery systems.

## Figures and Tables

**Table 1:** Drug loads and loading efficiency of Felodipine and Furosemide in mesoporous silica Syloid XDP 3050 (surface area of 310 m^2^/g).

% of Theoretical Surface Coverage	Felodipine-Syloid				Furosemide-Syloid			
	Theoretical Drug Load (%, g/g)	Actual Drug Load (%, g/g)	Normalised Actual Drug Load (×10^−3^ g/m^2^)	Loading Efficiency (%)	Theoretical Drug Load (%, g/g)	Actual Drug Load (%, g/g)	Normalised Actual Drug Load (×10^−3^ g/m^2^)	Loading Efficiency (%)
100%	12.6	12.5 ± 0.3	0.403 ± 0.010	99.2 ± 2.4	10.8	11.0 ± 0.9	0.355 ± 0.029	101.9 ± 8.3
200%	25.2	15.6 ± 0.2	0.503 ± 0.006	61.9 ± 0.8	21.6	16.6 ± 0.3	0.535 ± 0.010 *	76.9 ± 1.4
300%	37.8	21.6 ± 0.3	0.697 ± 0.010 *	57.4 ± 0.8	32.4	23.9 ± 0.5	0.771 ± 0.016	73.8 ± 1.5

(*): Drug load at which a complete amorphisation was successfully obtained.

**Table 2:** Drug load and loading efficiency of Felodipine and Furosimide in mesoporous silica Syloid XDP 3050 with the incorporation of HPMCAS.

% of Theoretical Surface Coverage	Felodipine-Syloid-HPMCAS			Furosemide-Syloid-HPMCAS		
	Theoretical Drug Load (%, g/g)	Actual Drug Load (%, g/g)	Loading Efficiency (%)	Theoretical Drug Load (%, g/g)	Actual Drug Load (%, g/g)	Loading Efficiency (%)
100%	12.6	13.1 ± 0.2	104.0 ± 1.6	10.8	10.7 ± 0.6	99.1 ± 5.5
200%	25.2	20.3 ± 0.5	80.6 ± 1.9	21.6	14.7 ± 1.3	68.1 ± 6.0
300%	37.8	30.3 ± 0.2	80.2 ± 0.5	32.4	23.8 ± 0.6	73.5 ± 1.9
